# A Systematic Review of Complementary and Alternative Veterinary Medicine in Sport and Companion Animals: Electrotherapy

**DOI:** 10.3390/ani13010064

**Published:** 2022-12-23

**Authors:** Heli K. Hyytiäinen, Anna Boström, Kjell Asplund, Anna Bergh

**Affiliations:** 1 Department of Equine and Small Animal Medicine, Faculty of Veterinary Medicine, University of Helsinki, P.O. Box 57, 00014 Helsinki, Finland; 2 Department of Public Health and Clinical Medicine, Umeå University, SE 901 87 Umeå, Sweden; 3 Department of Clinical Sciences, Swedish University of Agricultural Sciences, SE 750 07 Uppsala, Sweden

**Keywords:** electrotherapy, percutaneous electrical nerve stimulation, transcutaneous electrical stimulation, neural electrical muscle stimulation, pulsed electromagnetic field therapy, static magnet, bioelectricity, interference, sport and companion animals

## Abstract

**Simple Summary:**

Numerous electrotherapeutic methods are commonly used in complementary and alternative veterinary medicine, as well as in conventional veterinary medicine. In these methods, electrical currents are commonly used to affect nerves, muscles, bones, or other tissues. In a systematic literature review, we collected information from published articles on electrotherapies used in horses, dogs, and cats. After screening 5385 articles of potential interest, we identified 41 articles that contributed to answering the overriding question: What is the scientific evidence for electrotherapy in horses, dogs, and cats? For most of the therapies, the number of studies was low with small numbers of animals. Many of the studies were of insufficient scientific quality and the electrotherapy was applied in many different clinical conditions and therapeutic settings. This made it difficult to reach robust conclusions, except for one: no current electrotherapies have sufficiently strong scientific evidence to support clinical effects in the treatment of horses, dogs, or cats with conditions affecting muscles, joints, nerves, or bones. Based on limited promising results, we have listed some electrotherapies that could be evaluated in more detail in high-quality studies.

**Abstract:**

Electrotherapy modalities are currently used in the treatment of animals, but the evidence base supporting their use has not yet been systematically reviewed. Cochrane guidelines, as adapted by the Swedish Agency for Health Technology Assessment and Assessment of Social Services, were followed for this systematic review. A literature search regarding all currently known electrotherapy modalities applied to horses, dogs, and cats was conducted for the years 1980–2020 using three databases: CABI, PubMed, and Web of Science Core Collection. Of the 5385 references found, 41 articles were included in the review: 13 papers on pulsed electromagnetic field therapy (PEMFT), 7 on neural electrical muscle stimulation (NEMS), 5 on transcutaneous electrical nerve stimulation (TENS), 4 on static magnets, 3 on interference, 2 each on percutaneous electrical neural stimulation (PENS), bioelectricity, and diathermy, and 1 each on micro-pulsed stimulation, capacitive coupled electrical stimulation, and microwave therapy. The literature per modality was limited in quantity (mean 3.7 papers). Half of the articles were assessed to have a high risk of bias (20 high, 7 moderate, and 14 low). The existing literature used a spectrum of indications and treatment parameters, which makes comparisons and drawing conclusions to support the use of these modalities in clinical practice challenging. The current scientific evidence is not sufficient to support the clinical effects of electrotherapies for any clinical indication in horses, dogs or cats. The selected suggestive results warrant further high-quality research on PEMFT, NEMS, TENS, and PENS.

## 1. Introduction

Electrotherapy has various definitions and descriptions [1]. In this systematic review, the term stands for treatment using various types of electrical stimulation and electromagnetic radiation. Numerous electrotherapy modalities, such as pulsed electromagnetic field therapy (PEMFT), interference therapy, and diathermy [2,3,4], are examples of complementary and alternative veterinary medicine (CAVM) methods. However, some methods are commonly used in conventional medicine, for instance, transcutaneous electrical nerve stimulation (TENS) [5]. These methods can be used as part of the rehabilitation process or individually as single treatments. Professionals as well as lay people currently use electrotherapy modalities, as in many countries the methods’ availability and use on animals are not regulated by law [6,7,8,9,10]. Since electrotherapy equipment is often relatively affordable, and its selling or use is not controlled or restricted in any way, it is popular in CAVM. Often, this equipment is used by various paraprofessionals with varying educational backgrounds or by animal owners, with unknown insights regarding its use.

Despite the vast interest of the public in electrotherapy methods and the relatively common use of these methods among animal health professionals, evidence supporting their use in the treatment of sport and companion animals has not yet been systematically reviewed.

Veterinary medicine, as well as physiotherapy, should be evidence-based. Research-based knowledge enables clinicians to apply a treatment method to a patient safely and efficiently. In the treatment of animals, there are several ethical issues involved in the use of treatment modalities. Firstly, there is the potential risk of direct adverse reactions due to the unsafe use of a modality. Secondly, appropriate treatment may be delayed, thus increasing the unnecessary suffering of the animals due to use of ineffective treatment methods. Thirdly, the socioeconomic impact on the owner, in the form of unnecessary costs of time and money, should be considered, and fourthly the psychological stress due to worry over the animal’s well-being also warrants consideration. Thus, the use of these modalities should be based on scientific evidence.

This literature review aims to fill the void of systematically reviewed information regarding the use of electrotherapy modalities in the treatment of horses, dogs, and cats.

## 2. Materials and Methods

This systematic review was conducted according to the Cochrane guidelines [11], as adapted by the Swedish Agency for Health Technology Assessment and Assessment of Social Services (SBU) in its methodological handbook [12].

### 2.1. Review Topic/Research Question

The current scientific documentation for the clinical efficacy of available electrotherapy modalities used in sport and companion animals is assessed.

### 2.2. Search Strategy

A literature search was conducted by the library services of the Swedish University of Agricultural Sciences in November 2020. The three databases of CABI, PubMed, and Web of Science Core Collection were searched for available literature from 1980 to 2020.

The search words used were as follows: equine OR horse OR canine OR dog OR feline OR cat AND veterinary medicine OR veterinary, AND therapy* OR treatment*, AND neuromuscular electrical stimulation (NEMS) OR muscle stim* OR transcutaneous electrical nerve stimulation (TENS) OR percutaneous electrical neural stimulation (PENS) OR pulsed electromagnetic field (PEMFT) OR pulsed magnetic field OR electrotherapy OR functional electrical stimulation OR magnet therapy OR static magnet therapy OR electromagnetic therapy OR interferential therapy OR interferential current OR pulsed short-wave therapy OR H wave therapy OR diadynamic therapy OR high voltage pulsed current OR microcurrent therapy OR Russian stimulation OR burst mode alternating current OR iontophoresis. The references of the publications identified in the initial search by the librarians were imported to the Endnote (X9.3.3, 2018) reference management system, and shared among the authors.

### 2.3. General Inclusion and Exclusion Criteria

Original peer-reviewed journal publications with the subject species of equines, canines, or felines from the years 1980 to 2020 were included. In addition to observational and interventional clinical studies, experimental ones mimicking a clinical situation were included. Experimental studies reporting mechanism of action were excluded, as were reports including invasive methods (surgical implantation).

In accordance with the conventional conduct of systematic reviews, only original research articles with full text versions available were included. Abstracts, case reports (studies with less than five subjects), conference proceedings, opinion notes, review articles, double publications, and textbook chapters were excluded. To avoid confounding, studies where numerous interventions were performed simultaneously (i.e., concurrent TENS, non-steroidal anti-inflammatory drugs, and corticosteroid injections) were also excluded. The language of publication had to be English, Finnish, French, German, Italian, Portuguese, Spanish, or any of the Scandinavian languages. 

### 2.4. Study Selection and Categorization

For the following stages of the review process, one author (HH) applied the relevant inclusion and exclusion criteria to all publications.

In the first selection phase, articles of possible relevance for the review were identified based on the title and abstract of each publication. At this stage, duplicates and any publications with identified exclusion criteria were removed. Articles describing species other than horses, dogs, and cats were excluded

At the second selection phase, the full text of articles deemed potentially relevant to the review was accessed from available open access sources. Full texts that were not available in digital format were requested from the Swedish University of Agricultural Sciences library. If the full manuscript was not found during these steps, the publication was excluded. The final selection of the included articles was conducted based on the assessment of the full texts. 

For all included studies, the first author, year of publication, study design, study population, intervention, type of control, outcome, and relevance (external validity) were documented using a modified SBU template [12]. Assessment of the risk of bias (as a measure of scientific quality) of each article was performed in accordance with the Cochrane [11] and SBU [12] guidelines. The assessment was based on the following items: study design, statistical power, deviation from planned therapy, loss to follow-up, type of outcome assessment, and relevance. In the assessment of observational studies, risk of confounding factors was also included. To ensure consistency, prior to the literature review, three of the authors (ABe, HH, KA) reviewed a random sample of 12 papers independently, and the reviews were discussed to ensure standard assessment of the review process and categorization of the articles. After the final assessment of the articles included in this review by one of the authors (HH), another author (ABo) confirmed the assessment of each article. The writing of the paper has been conducted following the PRISMA 2022 checklist.

## 3. Results

### 3.1. Literature Selection Process

**Figure 1:**
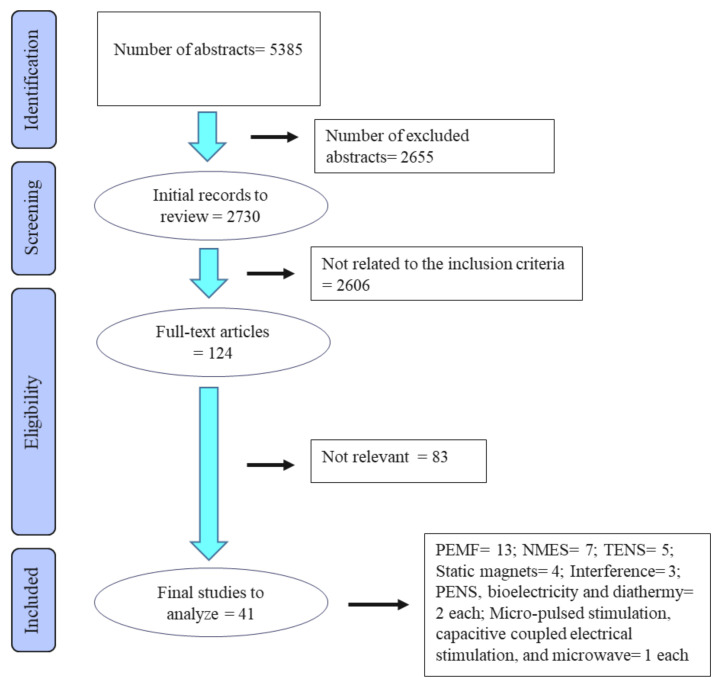
Flow diagram of the stages of the selection process used for identification of studies eligible for final analysis.

The primary literature search yielded 5385 references. After the first selection phase of the review process, 2730 references were further assessed. From these, 124 articles progressed to the eligibility stage of the selection process. After assessing the full texts of these articles, 41 articles remained to be included in the final review process. These 41 articles included 13 papers on PEMFT, 7 on neural electrical muscle stimulation (NEMS), 5 on TENS, 4 on static magnet, 3 on interference, 2 each on percutaneous electrical neural stimulation (PENS), bioelectricity and diathermy, and 1 each on micro-pulsed stimulation, capacitive coupled electrical stimulation, and microwave therapy (see Figure 1). The modalities are presented in Table 1.

### 3.2. Pulsed Electromagnetic Field Therapy

Thirteen publications regarding PEMFT were included in the review: three on horses [13,14,15], nine on dogs [3,16,17,18,19,20,21,22,23], and one on cats [24] (Table 2).

#### 3.2.1. Quality of Studies

There was one study with high, four with moderate, and eight with low risk of bias. Of these thirteen studies, information on power calculation was only stated in one [22]. One study had a significant number of animals lost to follow-up [21]. Some studies also presented small group sizes with the risk of chance findings [23,24]. In two studies with originally small groups, outcomes were measured in only some of the included animals at different time points, thus further decreasing the group size [16,19]. In some cases, the use of subjective outcome measures can also be considered a confounding factor [21]. 

Of the six randomized studies, three mentioned the method of randomization [3,14,22]. Blinding was stated in two studies [3,22]. Specific inclusion and exclusion criteria were provided in two of the thirteen studies [3,21]. In one study that did not provide the health status of animals, the omission may have affected the results [13].

#### 3.2.2. Clinical Indications

The clinical indications for horses were back pain [15], bone isotope uptake [13], and bone graft incorporation [14]. In dogs, the indications included thoracolumbar intervertebral disc extrusion [22], recovery from intervertebral disc disease-related hemilaminectomy [3], bone loss due to disuse [18], bone healing [17,20], osteoarthritis [21], cartilage growth and repair [23], and posterior spinal fusion [16,19]. In cats, the indication for the intervention was spinal cord injury [24].

#### 3.2.3. Interventions and Controls

PEMFT treatment parameters varied between the studies. Placebo treatment was used in six studies [3,14,15,18,20,23]. Three studies used controls that received no other treatment [16,19,24]. Three studies used the contralateral limb to the treated one, i.e., the same animal concurrently served as the intervention and the control subject [13,14,17].

#### 3.2.4. Clinical Effects

Five of the studies (mainly papers with low risk of bias) found PEMFT to have a positive effect on intervertebral disc disease and spinal cord injury-related recovery, osteotomy healing, osteoarthritis, and tissue-engineered cartilage grafts [3,20,21,23,24].

The positive outcomes included time to and quality of walking after paresis [24], new bone formation and resorption of the bone surface [18,20], and the enhancement of engineered cartilage growth and repair [23].

Two studies found PEMFT to be effective according to some but not all of outcome measures. PEMFT was related to improved bone cross-sectional area during disuse, improved proprioceptive placing, and a higher mechanical sensitivity threshold in relation to intravertebral disc extrusion [18,22]. Six of the studies (low-to-moderate bias) did not find any effect of PEMFT treatment [13,14,15,16,17,19]. No adverse reactions directly linked to PEMFT were reported.

**Table 2:** Review of articles regarding pulsed electromagnetic field therapy.

Study	Study Design	Control Group	Study Sample	Intervention and Dosage	Outcome Variables	Main Results	Study’s Risk of Bias
Alvarez et al. [3]	Randomized controlled clinical trial	A group receiving placebo treatment	28 + 27 = 53 dogs post-hemilaminectomy. Inclusion: Hemilaminectomy due to myelopathy following intravertebral disc disease. Non-ambulatory paraparesis or -plegia, neurologic grade > 3. Exclusion: prior episodes of intervertebral disc disease, masses or other spinal lesions, seizures, arrhythmias, concurrent conditions possibly affecting recovery, or unrelated medication.	PEMFT at 27.12 MHz, 2 ms pulse duration, 2 Hz, peak-induced magnetic field of 4 µT, 15 min every 6 h in hospital and every 12 h at home	Wound healing, pain, neurologic grading	Active PEMFT treatment group dogs’ wounds healed faster at six weeks, and they received less owner-administered pain medication during first seven days	Low
Biermann et al. [15]	Clinical placebo-controlled crossover study	-	20 horses (polo ponies in regular work) Inclusion: - Exclusion: -	Placebo or PEMFT blanket with ±50 mT, rectangular impulse, variable frequency 1–30 Hz, 40 min per day for 10 days	Algometry for mechanical nociceptive threshold, facilitated active flexion of the thoraco- lumbar spine with subjective grading	No improvement or change in comparison with control due to PEMFT treatment after 10 days of treatment	Low
Enzler et al. [17]	Experimental controlled trial	Contralateral limb of each dog was untreated and served as a control	12 dogs with midshaft osteotomies of both ulnas. Inclusion: - Exclusion: -	Ten 8 V and 175 µs pulse-trains per second, 24 h per day, 7 days a week for 22–30 days	Mechanical testing and radiology of the bones	No effect of the PEMFT treatment after the treatment period	Moderate
Collier et al. [13]	Experimental randomized controlled trial	Contralateral limb of each horse was untreated and served as a control	2 + 2 + 2 = 6 healthy horses Inclusion: - Exclusion: -	PEMFT with 30 G, 30 Hz (group 1) OR 99 G, 60 Hz (group 2) for four days, or two days of each (group 3), two coils on opposite sides of third metacarpal	Scintigraphy for isotope uptake on the bone, clinical observations (lameness)	No difference between the treated and control limbs’ metacarpal isotope uptake	Low
Crowe et al. [24]	Experimental randomized controlled trial	A group with no treatment	8 cats with induced spinal cord injury Inclusion: - Exclusion: -	PEMFT with pulse train 44, pulse width 100 µs, repetition rate 25 Hz, peak current 5 A, peak electric field 6.7 mV/cm, 4 h daily for 12 weeks on cats’ dorsal midline	Electrophysiology testing for somatosensory-evoked potentials, walking ability, histological examinations	PEMFT enhanced recovery of locomotor function in weekly observations, during a 12-week follow-up period	Low
Inoue et al. [20]	Experimental controlled trial	A group receiving placebo treatment	6 + 6 = 12 dogs with induced tibial osteotomy Inclusion: - Exclusion: -	PEMFT 30 s bursts of asymmetric pulses repeated at 1.5 Hz, with field rise 0–2 G in 230 µs and then returning to 0–30 µs, 1 h. Brace on the limb.	Weight-bearing analysis, radiographic analysis, biomechanical testing, histological and histomorphological analyses	In two-, four-, six-, eight-, ten- and twelve-week assessments: faster recovery of dynamic weight bearing, increased new bone formation, higher mechanical strength of osteotomy site	Low
Kahanovitz et al. [16]	Experimental controlled trial	A group with no treatment	5 + 5 = 10 adult dogs with induced lumbar spinal fusion. Inclusion: - Exclusion: -	PEMFT with repetitive bursts: primary pulse proportion quasi-rectangular slope 200 µs, positive peak amplitude of 1.1 mV/cm. Secondary pulse of opposite polarity, 28 µs, peak voltage 9.6 mV/cm. Asymmetric pulses for 5 ms, pulse groups repeated at rate 15 Hz for 12 h every day. Two coils in a body jacket.	Hematologic and serologic testing, histologic evaluation, radio-graphy	Early accelerated osteogenic response, but no histological or radiological differences between groups at and after 12 weeks. No improvement in overall results.	Moderate
Kahanovitz et al. [19]	Experimental controlled trial	A group receiving no treatment	8 + 8 + 8 = 24 dogs with induced lumbar facet fusions. Inclusion: - Exclusion: -	PEMFT with pulse burst frequency 1.5 Hz, pulse burst duration 30 ms, individual pulse duration 260 µs, positive excursion 1 G, negative 0/15 G; 30 min (group 1) or 60 min (group 2) for 12 weeks.	Radiography, histology	No differences between the groups at 6- or 12-week evaluation points	Moderate
Kold et al. [14]	Experimental randomized controlled trial	Contralateral limb served as a placebo control; A group receiving no treatments.	2 + 6 = 8 yearling ponies with metacarpal cancellous bone crafts. Inclusion: - Exclusion: -	PEMFT 3 h/day, asymmetric pulse burst 30 ms duration repeated at 1.5 Hz, each pulse 250 µs positive of 2.4 mV peak, 14µs negative of −130 mV peak, repeated 155 times in each burst. Coils medially and laterally to the metacarpal bones.	Radiography, quantitative assessment of graft incorporation	At the assessment points, between 9 to 241 days after installation of the craft, no significant benefit of the PEMFT treatment radiologically, and minor benefit of graft incorporation	Low
Pinna et al. [21]	Randomized controlled clinical trial	A group receiving medical treatment	15 + 25 = 40 dogs with osteoarthritis in one or more joints. Inclusion: lame at least 4 weeks, radiographically diagnosed osteoarthritis in at least one joint, osteoarthritis as cause for lameness. Exclusion: systemic diseases, infectious arthritis, neurological or other osteoarthritis orthopedic disease, non-steroidal analgesic drugs, corticosteroids or opioids during the past two weeks, pregnancy.	PEMFT with quantum resonance system to whole body via a treatment mat, cyclic frequency 3–22–250–500 Hz, intensity 0.75 µT, 10 min + treatment pad on joint, cyclic frequency of 0.3–1.5–3 Hz, intensity of 0.75 µT for 8 min, 3–6 times per week, 20 times	Lameness, pain on palpation, range of motion, radiographic osteoarthritis, chronic pain, owner satisfaction	PEMFT treatment group dogs’ signs improved during the study period (measured at 10 and 20 days, 4 and 12 months after the start of therapy)	High
Skerry et al. [18]	Experimental controlled trial	A group receiving placebo treatment	9 + 8 = 17 dogs with disuse induced by proximal and distal osteotomies. Inclusion: - Exclusion: -	1.5 Hz repetitions of 30 ms bursts of asymmetric pulses, varying from + 2.5 to −135 Hz/mV, 1 h per day, 5 days per week. Coil laterally on the fibula.	Measurements of cross-sectional area, frequency of intracortical remodeling events, and bone formation rate in secondary osteons, degree of osteonal closure, surface modeling/remodeling	PEMFT was related to less reduction in cross- sectional area, but not in the other parameters after 12 weeks of treatment	Low
Stefani et al. [23]	Experimental controlled trial	A group receiving placebo treatment	4 + 4 = 8 dogs with allogenic osteo-chondral constructs Inclusion: - Exclusion: -	Placebo (group 1) and PEMFT device (group 2) worn on the operated limb, with 1.5 ± 0.2 mT magnitude pulse, duration 1.3 ms and frequency of 75 Hz, duty cycle of 0.10. Used for 6 h/day, 7 days/week for 3 months.	Clinical lameness, functional gait, comfortable range of motion, pain, total pressure index, histology, and osteo-arthritic changes	Greater likelihood of normal chondrocyte and proteoglycan histological scores after 3 months	Moderate
Zidan et al. [22]	Randomized controlled clinical trial	A group receiving placebo treatment	8 + 8 = 16 dogs with surgically treated intervertebral disc extrusion. Inclusion: <20 kg, 2–12 years, paraplegia with no pain perception in either limb or tail, <2 days non-ambulatory, localization T3-L3, dg. acute thoracolumbar intervertebral disc disease. Exclusion: systemic comorbidity which might affect recovery, progressive myelomalacia, adverse behavior.	27.12 MHz carrier, burst 2 ms, 0.05 G; 15 min on, 2 h off; treatment loop in a jacket for 2 weeks, then manual loop twice daily for 4 weeks	Open field gait scores, ability to ambulate, time to independent ambulation, proprioceptive placing, hopping reaction, pain perception, mechanical sensory threshold (algometer), patellar and withdrawal reflexes, voluntary urination, bladder volume after urination	At 6 weeks follow-up, proprioceptive placing and higher mechanical sensitivity threshold were seen in dogs that had received PEMFT treatment	Low

(Pulsed electromagnetic field therapy = PEMFT; hertz = Hz; megahertz = MHz; second = s; microseconds = µs; milliseconds = ms; volts = V; millivolts = mV; microtesla = µT; millitesla = mT; gauss = G; minutes = min; hours = h; none = -).

### 3.3. Neural Electrical Muscle Stimulation

Five studies on horses [25,26,27,28,29] and two studies on dogs [30,31] were included in the review (Table 3).

#### 3.3.1. Quality of Studies

There were six studies with high and one study with low risk of bias. None of the papers reported deviation from planned therapy, or power or sample size calculations. Only one paper reported blinding (laboratory personnel blinded for intervention/control samples) [28]. In one of the studies, a significant number of patients had been lost to follow-up [26]. The remaining six papers did not report any patients lost to follow-up. None of the studies reported inclusion or exclusion criteria. Most of the studies, except for the one by Bergh et al. [25], had a high bias due to several confounding factors in their methodology. The clinical assessment of animals’ health was subjective in three studies [26,27,28]. Lack of specific diagnoses can be considered a confounding factor in four of the seven studies [26,27,28,29]. One study presented only descriptive data, without statistics, with a large variation in follow-up times and in the number of treatment times [26]. The only randomized study did not explain the method of randomization and did not use a control group [31]. In one study where a control group was used (non-randomized), comparisons between the intervention and control groups before and after the treatment were not made [30].

#### 3.3.2. Clinical Indications

The outcomes of the studies were possible changes in fiber types and physiological factors of equine muscles [25,27,28], muscle strength and hypertrophy [29], muscle spasm and hypertonicity [26], and (induced) muscular atrophy in dogs [30,31].

#### 3.3.3. Interventions and Controls

The intervention method was NMES in all of the studies. Only two of the seven studies used controls: one using a true control group with no treatment and the other a crossover design [25,30]. The remaining five studies did not have control groups.

#### 3.3.4. Clinical Effects

The Bergh et al. [25] study had a low risk of bias and did not find significant results in the outcomes assessed. The other studies, although reporting mainly positive results, such as improved muscle force and fiber size, had a high risk of bias.

**Table 3:** Review of articles regarding neural electrical muscle stimulation.

Study	Study Design	Control Group	Study Sample	Intervention and Dosage	Outcome Variables	Main Results	Study’s Risk of Bias
Bergh et al. [25]	Prospective crossover study	-	6 healthy horses Inclusion: - Exclusion: -	NEMS with biphasic rectangular pulse, frequency 50 Hz, pulse width 300 µs, 3 s ramp up, 2 s down, 10 s full contraction. Individual current amount. Conducted once a day for 4 weeks, with amount of contractions per week 1: 3 times 10; week 2 and 3: 3 times 15; week 4: 3 times 20. Two electrodes per muscle on motor points.	Muscle biopsy, histochemical analysis, biochemical analysis	No significant differences between the control side and the baseline on the stimulated side at 4-week follow-up	Low
Hernandez-Fernandez et al. [29]	Observational cohort study	-	5 horses Inclusion: - Exclusion: -	Bidirectional rectangular current, frequency 40 Hz, pulse width 200 µs, 15/30 s on/off time. Duration 15 min, adding 5 min every 2 weeks, max 30 min. Intensity increased until strong thoracic flexion. Five times per week for 12 weeks.	Thoracic flexion induced manually, with NEMS and EMG needle, during which muscle activity was measured with EMG	NEMS training increased muscle force and fatigue resistance at 12 weeks	High
Pelizzari et al. [30]	Experimental controlled trial	A group with no treatment	4 + 4 = 8 dogs with induced mm. Quadriceps atrophy. Inclusion: - Exclusion: -	NEMS with 2500 Hz, pulse duration of 50%, 3 s ramp up and down, 12/25 s on/off time, 30 min. Conducted every 48 h (3 times a week) for 60 days.	Thigh peri- metry, stifle range of motion, creatine kinase, morphometry of vastus lateralis muscle fibers	Stifle range of motion increased significantly between baseline and 30 days in the TENS group. Mm. vastus lateralis fiber transversal area at day 90 had improved in the TENS group.	High
Pelizzari et al. [31]	Experimental randomized trial	-	4 + 4 = 8 dogs with induced mm. Quadriceps atrophy Inclusion: - Exclusion: -	NEMS with 2500 Hz, pulse duration of 50%, 3 s ramp up and down, 12/25 s on/off time. Conducted every 48 h (3 times a week) for 60 days for 30 (group 1) or 60 (group 2) min at a time.	Thigh perimetry, stifle range of motion, creatine kinase, morphometry of vastus lateralis muscle fibers	Increase in range of motion of the stifle at day 30 as well as the vastus lateralis fiber transversal area at day 90 in comparison to baseline, with 60 min intervention having more effect	High
Ravara et al. [27]	Prospective cohort study	-	6 horses with owner reported back pain Inclusion: - Exclusion: -	FES with pulsed, biphasic, rectangular waveform at 60 Hz, with a 0 net charge, pulsed 2/2 s on/off, voltage between 7.6 and 15.8 V for 35 min. Six electrodes in a pad were used three times per week for 8 weeks = 22 times.	Modified Ashworth Scale for muscle spasm, muscle biopsies	One-grade decrease in muscle spasm after 4 treatments. Muscle fiber size increased in a few horses; only one horse had long-term denervated muscle fibers post-FES; increased density and distribution of mitochondria indicated at eight weeks after the start of the treatment.	High
Schils, Turner [26]	Retrospective cohort	-	241 horses with epaxial muscle problems Inclusion: - Exclusion: -	FES with pulsed biphasic rectangular waveform, frequency 60 Hz, voltage ranging between 3.8 and 11 V, 35 min. Six electrodes in a pad on T10-L2 or L1-S5.	Modified Ashworth Scale for level of grade of spasm	Most horses’ spasm reduced by a grade after two treatments	High
Schils et al. [28]	Prospective cohort study	-	6 horses with owner reported back pain Inclusion: - Exclusion: -	FES with pulsed, biphasic, rectangular waveform at 60 Hz, with a 0 net charge, pulsed 2/2 s on/off, voltage between 7.6 and 15.8 V for 35 min. Six electrodes in a pad were used three times per week for 8 weeks = 22 times.	Muscle biopsies	A positive effect on mitochondrial density and distribution in muscle fibers at eight weeks after the start of the treatment	High

(Neural electrical muscle stimulation = NEMS; functional electrical stimulation = FES; hertz = Hz; microseconds = µs; milliamperes = mA; second = s; millisecond = ms; minutes = min; thoracic spine = T; lumbar spine = L; none = -).

### 3.4. Transcutaneous Electrical Nerve Stimulation

Five studies, three in dogs [32,33,34] and two in horses [5,35], were included in the review (Table 4).

#### 3.4.1. Quality of Studies

There were three studies with high and two studies with moderate risk of bias. All except one [35] of the studies were recent (<10 years) controlled studies. None of the papers reported power or sample size calculations, although significant results were reported in all five papers: four in favor of TENS intervention and one in favor of other compared interventions [18]. No deviations from planned therapy or significant loss to follow-up were reported in any of the studies. Selection bias could not be assessed, as the recruiting or status of the animals was reported in only one of the papers [35]. Moreover, the assessors of outcome were not blinded to the treatment in any of the papers. All except one study [5] used partly or subjective non-validated outcome measures to assess their treatment effect, whereas one of the studies did not report their outcome measures at all [32]. The only randomized control study did not describe their method of randomization [32].

#### 3.4.2. Clinical Indications

Clinical indications were sciatic nerve injury and ankylosing spondylitis in dogs [32,33,34], and superficial flexor tendon injury and epaxial muscle pain in horses [5,35].

#### 3.4.3. Interventions and Controls

The results of the TENS treatment were compared with a control group in each of the five studies, but placebo control was not used in any of the studies, and only three of the studies used a control group with no other treatments [5,32,34]. Two studies had only two control groups with other interventions performed on both [33,35].

#### 3.4.4. Clinical Effects

Both Sharifi et al. [32] and Srivastava et al. [34] reported similar positive outcomes for sciatic nerve-injured dogs with TENS treatment. The results indicated faster improvement in the TENS group in than in the control group. Due to several confounding factors in both of studies, they had a moderate-to-high risk of bias. The Krstic et al. [33] study on ankylotic spondylosis in dogs also showed a high risk of bias and reported positive effects from TENS treatment in some pain-related outcome variables compared with other treatments. In the Sharifi et al. [5] study, an objective mechanical measure of tendon tensile strength was used, revealing the difference between the TENS-treated group in relation to the control group, albeit the TENS-treated tendon was not equivalent to the normal tendon at the time of the final examination. This study had a moderate level of risk of bias. Mercado et al. [35] reported in their study with a high risk of bias that traumatic myositis-related back pain persisted after 30 days of treatment.

**Table 4:** Review of articles regarding transcutaneous electrical stimulation.

Study	Study Design	Control Group	Study Sample	Intervention and Dosage	Outcome Variables	Main Results	Study’s Risk of Bias
Krstic et al. [33]	Controlled clinical trial	A group receiving interference and a group receiving microwaves	8 + 8 + 8 = 24 dogs with ankylosing spondylitis Inclusion: - Exclusion: -	TENS with symmetrical impulses at 85 Hz, impulse width of 0.4 ms (increased with 0.1 ms every other day until 1 ms), current of 7 mA; 12 s contraction, 24 s relaxation *, 15 min daily, for 10 days	Pain assessed by owner and vet with a questionnaire and VAS, heart and respiratory rate, lameness, atrophy, pain in hip movement	After ten days of treatment pain reduced during rest and activity, back palpation and hip ROM	High
Mercado et al. [35]	Controlled clinical trial	A group receiving ultrasound and a group receiving TENS and ultrasound	63 horses with mm. longissimus dorsi myositis Inclusion: - Exclusion: -	TENS with bipolar technique, 50 cycles per s, intensity of 2 mA for one hour, 3 times per day for 30 days	Clinical estimation, ultrasonography	Animals still showed signs of pain and mild fibrosis in the area at 28 days of follow-up	High
Sharifi et al. [5]	Experimental controlled trial	A group receiving no treatment	4 + 4 = 8 horses with induced front limb superficial digital flexor tendon split. Inclusion: - Exclusion: -	TENS with 100 Hz frequency, intensity of 80 µs, 10 min daily for 14 days	Tensile strength of SDFT tendon after an induced lesion	Significantly better tensile strength in TENS-treated tendons 60 days post-trauma	Moderate
Sharifi et al. [32]	Experimental randomized controlled trial	A group with electroacupuncture and a group with no treatment	5 + 5 + 5 = 15 dogs with crushed sciatic nerve Inclusion: - Exclusion: -	TENS with four electrodes, frequency of 100 Hz, intensity of 80 µs (2.8 ± 1.6 mA), 10 min daily	Weight-bearing and EMG activity	Pain and swelling reduced, full weight bearing in four to five weeks. EMG activity in mm. semimembranosus and semitendinosus improved significantly in comparison to controls.	Moderate
Srivastava et al. [34]	Experimental controlled trial	A group with no treatments	5 + 5 = 10 dogs with crushed sciatic nerve Inclusion: - Exclusion: -	TENS with frequency of 100 Hz, intensity of 80 µs, 10 min daily for 15 days		Normal weight bearing and limb coordination during 5^th^ week, unlike the control group	High

(Transcutaneous electrical nerve stimulation = TENS; hertz = Hz; microseconds = µs; milliamperes = mA; second = s; millisecond = ms; minutes = min; visual analogue scale = VAS; none = -) (* Note: authors have reported this study as regarding TENS, but seem to have used an NEMS protocol).

### 3.5. Static Magnet

Four studies, two on horses [36,37] and two on dogs [38,39], were included in the review. All studies were randomized controlled trials, and one had a crossover design [36] (Table 5).

#### 3.5.1. Quality of Studies

There were three studies with low and one study with moderate risk of bias. All four studies were randomized, but only Edner et al. (2015) describe the actual randomization protocol used. Blinding was stated and described in all but one study [39]. In one of the studies [36], there was a 30% loss of animals from recruitment to data analysis due to data corruption. The other three studies did not report any animals lost to follow-up. None of the studies described power analysis or sample size calculation. None of the studies presented inclusion or exclusion criteria.

#### 3.5.2. Clinical Indications

The expected outcomes in horses were physiological changes in blood flow [36,37], muscle tension, and skin temperature [36]. In dogs, the indications were bone healing [39] and osteoarthritis [38].

#### 3.5.3. Interventions and Controls

Magnetic blankets [36], magnetic wraps [37,39], and magnetic mattresses [38] were used as interventions. Placebo control groups were used in all but one study. The one exception was the use of the contralateral limb of each animal as a control [37].

#### 3.5.4. Clinical Effects

No adverse reactions to the treatment were reported in any of the studies. Only one of the studies reported a confidently positive effect due to the treatment [39]. One study reported a cautiously positive trend [40], and two reported no effects related to the intervention [36,37].

**Table 5:** Review of articles regarding static magnet.

Study	Study Design	Control Group	Study Sample	Intervention and Dosage	Outcome Variables	Main Results	Study’s Risk of Bias
Edner at al. [36]	Prospective crossover study	-	10 healthy horses Inclusion: - Exclusion: -	Blanket with 120 unipolar 2.5 cm 900 G static magnets for 60 min	Muscle blood flow, skin temperature, pressure algometer for mechanical nociceptive threshold, behavior with ethograms	Magnet blanket did not have an effect on the assessed parameters during treatment or 30 min after it	Low
Rogachefsky et al. [38]	Experimental randomized controlled trial	A group with no mattresses or magnets. A group with placebo mattresses. A group with magnet mattresses (unaffected limbs of dogs from the above listed groups).	6 + 6 + 6 = 18 dogs with induced stifle osteoarthritis Inclusion: - Exclusion: -	Cage mattress with 72 magnets with 1100 G (0.11 T), field strength of 450–500 G (45–50 mT) for 12 weeks	Synovial effusion, gross and microscopic anatomy of the stifle and cartilage structure, microscopic anatomy, immunohistochemical studies, Western blot analysis	Exposure to the magnetic field could be related to less severe anatomical changes in the cartilage at 12 weeks	Low
Saifzadeh et al. [39]	Experimental randomized controlled trial	A group of dogs with placebo treatment	5 + 5 + 5 = 15 dogs with osteotomy of midshaft of radius Inclusion: - Exclusion: -	Magnetic wraps with either 700 G (group 1) or 1500 G (group 2) static magnetic field for 8 weeks	Subjective lameness score, breakability of the osteotomized radius	1500 G group dogs had better improvement in the lameness score and a higher breakability threshold of the osteotomized radius than the other groups at eight weeks	Moderate
Steyn et al. [37]	Randomized controlled clinical trial	Placebo treatment on the contralateral limb of each animal	6 healthy horses Inclusion: - Exclusion: -	Magnetic wrap with 27 Gs for 48 h	Scintigraphy for relative perfusion ratio	Intervention does not increase blood flow in the area under the wrap after 48 h treatment	Low

(Gauss = G; tesla = T; minutes = min; hours = h; none = -).

### 3.6. Interference

Three studies were identified to meet the inclusion criteria for the review, all on dogs [2,33,40] (Table 6).

#### 3.6.1. Quality of Studies

There were two studies with high and one with low risk of bias. All studies were controlled studies, with one [2] being randomized, although the method of randomization was not revealed. No deviations from planned therapy or significant loss to follow-up were reported. None of the papers reported power or sample size calculations. Selection bias could not be assessed, as the recruiting or health status of the animals was not reported. In one of the studies [40], the lack of specific diagnosis related to the inclusion criteria (hindquarter weakness) presented a significant confounding factor to the study. Moreover, only in one of the papers [2] was the blinding of assessors described. Subjective and non-validated outcome measures were used [33].

#### 3.6.2. Clinical Indications

Indications were osteoarthritis [2], ankylosing spondylitis [33], and hindquarter weakness [40].

#### 3.6.3. Interventions and Controls

One study had two control groups, with other interventions performed in both [33]. One study had a control group with the same basic treatment in all groups and a control group with another therapy method [40]. Only one study used placebo as a control [2].

#### 3.6.4. Clinical Effects

Muscle atrophy was reported to have decreased during the 10-day follow-up period in the Krstic et al. [33] study, but which muscles and the type of assessment were not described. The study had a high risk of bias. Upariputti et al. [2] found that interference treatment increased the peak vertical force in dogs with coxofemoral osteoarthritis compared with controls, thus indicating an analgesic effect. This study had a low risk of bias. Maiti et al. [40], in their high-risk bias study, summarized their findings of numerous outcome measures, with the interference group showing the most recovery.

**Table 6:** Review of articles regarding interference therapy.

Study	Study Design	Control Group	Study Sample	Intervention and Dosage	Outcome Variables	Main Results	Study’s Risk of Bias
Krstic et al. [33]	Controlled cohort trial	A group receiving TENS and a group receiving microwaves	8 + 8 + 8 = 24 dogs with ankylosing spondylitis Inclusion: - Exclusion: -	Interference with one electrode pair at constant 4000 Hz, other at variable 3850–4000 Hz, with dynamic change in current intensity 30 mA ± 10%, change in frequency of 50 Hz to 150 Hz and back; 15 min for 10 days. Lesion in the crossing of currents.	Pain assessed by owner and vet with a questionnaire and VAS, heart and respiratory rate, lameness, atrophy, hip movement	After 10 days of treatment pain reduced during rest and activity, back palpation and atrophy, and increased hip range of motion	High
Maiti et al. [40]	Controlled cohort trial	A group receiving conventional therapy and a group receiving ultrasound therapy	5 + 5 + 5 = 15 dogs with hind quarter neuromuscular disorders. Inclusion: Dog can stand and has staggering gait but intact pain sensation. Exclusion: -	Interferential therapy with base frequency of 100 Hz, spectrum frequency 50 and program number 12 for 10 min three times a week	Mental status, general conditions, clinical signs, respiration and heart rates, rectal temperature, neurological examination, hemoglobin, packed cell volume, total leucocyte count, differential leucocyte count, total protein, glucose, alkaline phosphatase activity	Of the three groups, the interferential groups showed maximal recovery	High
Upariputti et al. [2]	Randomized placebo-controlled cross-over study	A group with placebo treatment and a group without treatment	9 dogs with coxofemoral osteoarthritis. Inclusion: Healthy dogs with clinical evidence of coxofemoral osteoarthritis, either gender, over 2 years of age, weighing over 15 kg Exclusion: -	Carrier frequency 4 kHz, AMF 100 Hz, pulse duration 250 µs, Four electrodes placed around the coxofemoral joint.	Lameness score, articular mobility score, articular pain score, ground reaction force measurement	After single treatment: no change in lameness, articular mobility, and articular pain scores, and peak vertical force increased	Low

(Hertz = Hz; microseconds = µ; milliamperes = mA; VAS = visual analogue scale; none = -).

### 3.7. Percutaneous Electrical Neural Stimulation

Two descriptive case series studies were included for analysis in the PENS-related literature review [41,42] (Table 7).

#### 3.7.1. Quality of Studies 

Both reviewed studies were recent (<10 years) but were considered to have a high bias mainly due to the reports’ study design, with a convenience sample, lack of control groups, low statistical power, and inferior statistical analysis [42]. Length of follow-up varied between animals [42]. In addition, the assessors were aware of the intervention undertaken, the treatment itself was not fully standardized, and approximately 15% of animals were lost to follow-up in one of the two studies [41].

#### 3.7.2. Clinical Indications

The clinical indication for both articles was trigeminal-mediated headshaking syndrome in horses.

#### 3.7.3. Interventions and Controls

PENS was used as a treatment in both studies, and there were no controls in either one.

#### 3.7.4. Clinical Effects

Varying improvement in symptoms was reported, with mainly positive outcomes, i.e., most of the horses seemed to benefit from the PENS treatment when judged by the severity and existence of their clinical signs (head shaking). Some adverse effects, such as hematoma on the treatment site and the brief aggravation of signs, were also reported in some cases.

**Table 7:** Review of articles regarding percutaneous electrical stimulation.

Study	Study Design	Control Group	Study Sample	Intervention and Dosage	Outcome Variables	Main Results	Study’s Risk of Bias
Roberts et al. [42]	Descriptive case series	-	7 horses with trigeminal-mediated head shaking. Inclusion: clinical signs of trigeminal-mediated headshaking at the time of study, no seasonal remission. Exclusion: previous coil compression treatment	PENS, 2 Hz and 100 Hz with 3 s alternations, voltages ranging between 0.2 and 2.7 V for 25 min/repeated when clinical signs reoccur	Return to riding use	Most horses benefit from PENS and return to ridden work for varying amounts of time	High
Roberts et al. [41]	Clinical cohort study	-	168 horses with trigeminal-mediated head shaking. Inclusion: trigeminal-mediated headshaking diagnosis, no other treatment at the time of the study, ridden/lunged prior to start of signs. Exclusion: -	No parameters provided. Three treatments were conducted: the 2nd 3–7 days and the 3rd 10–14 days after the previous treatment.	Return to previous level of performance (being ridden)	Approximately half of the horses benefitted from PENS, and half did not respond	High

(Percutaneous electrical neural stimulation = PENS; hertz = Hz; volts = V; none = -).

### 3.8. Bioelectricity

Two case series, one on small animals [43] and one on horses [44], were included in the review (Table 8).

#### 3.8.1. Quality of Studies

Being case series reports with low numbers of animals (4 dogs, 1 cat, 10 horses), the reports had several severe confounding factors such as varying reasons for wounds and differences in wound management and start of treatment time in relation to wound healing. Moreover, in one of the two studies, there were no actual outcome measurements conducted, only “estimations” [43]. Therefore, due to the nature of the case studies, both reports had a high risk of bias.

#### 3.8.2. Clinical Indications

Wounds were the indication in both included studies.

#### 3.8.3. Interventions and Controls

Bioelectric treatment was the intervention in both studies. There were no controls in either study.

#### 3.8.4. Clinical Effects

Reported wounds healed in both studies. Neither study stated adverse effects of the treatment.

**Table 8:** Review of articles regarding bioelectricity.

Study	Study Design	Control Group	Study Sample	Intervention and Dosage	Outcome Variables	Main Results	Study’s Risk of Bias
Maijer et al. [43]	Case series	-	4 dogs + 1 cat with traumatic wounds. Inclusion: Single complex wound with one or more complications. Exclusion: Free of serious comorbidities.	Bioelectric dressing changed at 6 h to 8 days, for 1–4 weeks	Wound healing estimated by clinician	All wounds healed in 1–4 weeks, with no complications or patient discomfort	High
Varhus et al. [44]	Case series	-	10 horses with varying types of wounds Inclusion: - Exclusion: -	Bioelectric dressing changed every 3 to 4 days	Wound dimension measurement and photography	Method is safe and effective in facilitating healing of acute and chronic wounds	High

(None = -).

### 3.9. Diathermy

Two studies on short-wave diathermy fulfilled the inclusion criteria for this review, and both were conducted on dogs [4,45] (Table 9).

#### 3.9.1. Quality of Studies

There were two studies with high risk of bias. No significant numbers of dogs were lost to follow-up, and no deviations from planned therapy were reported in either study. Despite the studies being randomized and controlled, they were considered to have a high risk of bias. The main reason was varying background and history of pathology as well as the pathology itself (hindquarter weakness for undefined and undiagnosed reasons), in the relatively small study groups. Lack of clear inclusion and exclusion criteria, lack of power and sample size calculations, and lack of blinding and information regarding the method of randomization contributed to the risk of bias. The two studies shared the same confounding factors.

#### 3.9.2. Clinical Indications

The indication in both studies was hindquarter weakness [4,45].

#### 3.9.3. Interventions and Controls

In both studies, there were three groups with similar varying interventions. One group received diathermy, one conventional drug therapy, and one ultrasound and drug therapy.

#### 3.9.4. Clinical Effects

Diathermy was reported to be beneficial in reducing antioxidant stress [45], and to lead to better recovery in dogs with hindquarter weakness [4].

**Table 9:** Review of articles regarding diathermy.

Study	Study Design	Control Group	Study Sample	Intervention and Dosage	Outcome Variables	Main Results	Study’s Risk of Bias
Ansari et al. [4]	Randomized controlled trial	A group receiving conventional drug therapy and a group receiving ultrasound therapy in addition to conventional drug therapy	8 + 8 + 8 = 24 dogs with hind quarter weakness Inclusion: - Exclusion: -	Conventional drug therapy and shortwave diathermy with 200 mA intensity, 9 V AC, 10 min daily for 14 days; 2 pads on lumbar region.	Rectal temperature, heart and respiratory rate, different neurological parameters	During 28-day follow-up period, dogs treated with short-wave diathermy recovered sooner and better than the dogs in the other groups	High
Zama et al. [45]	Randomized controlled trial	A group receiving conventional drug therapy and a group receiving ultrasound therapy in addition to conventional drug therapy	8 + 8 + 8 = 24 dogs with hind quarter weakness. Inclusion: - Exclusion: -	Conventional drug therapy and shortwave diathermy with 200 mA intensity, 9 V AC, 10 min daily for 14 days; 2 pads on lumbar region.	Blood samples for oxidant-antioxidant balance	Outcomes were measured at days 3, 7, 14 and 28. Dogs treated with shortwave diathermy had more reduced oxidative stress than the ones in other groups.	High

(Milliamperes = mA; volts = V; alternating current = AC; minutes = min; none = -).

### 3.10. Single Publications of Electrotherapy Modalities

Only one study of each of the following modalities met the inclusion criteria for the review: micro-pulse stimulation [46], capacitive coupled electrical stimulation [47], and microwave therapy [33]. One of the studies was on horses [46] and the other two were on dogs (Table 10).

#### 3.10.1. Quality of Studies

There were two studies with high and one study with low risk of bias. No significant numbers of dogs were lost to follow-up, nor were deviations from planned therapy reported in any of the studies. Power or sample size calculations were not reported; small numbers of animals were likely used in all studies. Selection bias could not be assessed, as the recruiting status of the animals was not reported thoroughly in any of the papers. Blinding was also not reported. Only one of the papers stated any inclusion criteria [46]. Further study-specific confounders included lack of clear diagnosis, control group, and statistical analysis [46], use of non-validated outcome measures, neglect of the effect of other consecutive treatments on the results, and lack of an untreated control group [33].

#### 3.10.2. Clinical Indications

The clinical indications included the following: strength of regenerating bone [47], swelling [46], and canine ankylosing spondylitis [33].

#### 3.10.3. Interventions and Controls

Interventions and control groups were as follows: micro-pulsed stimulation with no controls, capacitive coupled electrical stimulation with a control group receiving no treatment, and microwave therapy with control groups receiving other treatments.

#### 3.10.4. Clinical Effects

Microwave therapy was reported to be beneficial in some of the evaluated pain and mobility-related outcome measurements [33]. Micro-pulsed stimulation was reported to be beneficial in swelling reduction [46]. In the applied manner, capacitive coupled electrical stimulation was found to hinder the recovery of bone [47].

**Table 10:** Review of articles regarding single publications of electrotherapy.

Study	Study Design	Control Group	Study Sample	Intervention and Dosage	Outcome Variables	Main Results	Study’s Risk of Bias
Microwave							
Krstic et al. [33]	Controlled clinical trial	A group receiving TENS A group receiving Interference	24 dogs with ankylosing spondylitis Inclusion: - Exclusion: -	Microwave: radiator placed 5–10 cm from body surface, output 40 W, 2450 ± 50 Hz, wavelength of 12.2 cm. Daily 15 min, for 10 days	Pain assessed by owner and vet with a questionnaire and VAS, heart and respiratory rate, lameness, atrophy, and hip movement	After 10 days of treatment, pain reduced during rest and activity, back palpation and increased hip range of motion	High
Micro-pulse stimulation							
Nedvedova et al. [46]	Case series	-	9 horses with swelling of the limb. Inclusion: Swelling caused by minor injuries. Exclusion: -	Once a day <30 min. No parameters provided.	Subjective assessment of swelling reduction, mobility, soreness, wound healing	Micro-pulse stimulation has positive effect on swelling reduction	High
Capacitive coupled electrical stimulation							
Pepper et al. [47]	Experimental controlled trial	A group with no stimulation	17 + 17 = 34 dogs with experimental tibial lengthening procedure Inclusion: - Exclusion: -	Capacitive coupled electrical stimulation 60 kHz ± 10% sinusoidal-shaped wave with peak-to-peak voltage 3–6.3 V, output current of 5–10 mA root mean square	Radiographs, mechanical, histological, and histomorphometrical tests	After 28 days of treatment, recovery of bone strength was delayed	Low

(Hertz = Hz; kilo herz = kHz; microseconds = µ; milliamperes = ma; volts = V; watts = W; minutes = min; centimeters = cm; none = -).

## 4. Discussion

Regarding the electrotherapy treatment methods reviewed in this paper, it is obvious that the amount of evidence is very limited. There were, on average, 3.7 papers per modality (range 1–13, median 6). Most of the papers (21 of 41) were recent, i.e., less than 10 years old. Thirteen were 10–20 years old, three 20–30 years old, and four 30–40 years old.

Use of terminology seemed to be very challenging in the selection of publications. Firstly, some papers inappropriately referred to an electrotherapy modality as “physiotherapy”. We note that a single modality should not be named as equivalent to physiotherapy, which is an entity of various methods [48].

Secondly, there was contradictory and partly misleading use of nomenclature regarding the modalities. To clarify, all electrical treatments where electricity is delivered to the surface of the skin are essentially transcutaneous electrical stimulation. This includes several individual modalities, such as NEMS, interference, microcurrent, etc. These methods also include TENS, a modality named after the description of the method.

The term PENS, by contrast, means percutaneous electrical neural stimulation, and as such, includes all types of treatments and machines, where skin-penetrating electrodes are used for delivering electricity to the target tissue. For example, electroacupuncture is a form of PENS. Moreover, neural electrical muscle stimulation can be achieved with any type of machine that stimulates motor function with electricity. NEMS and “Muscle stimulator” are common names for machinery used primarily for this purpose. In a few papers reviewed here [26,27,28], FES was presented as its own method, when it is actually an NEMS method, and they are both essentially TENS methods. A paper reported having studied TENS [33], albeit using electricity to cause muscle contraction with unequal contraction and relaxation sequences. Generally, this would be perceived as NEMS, not TENS. The use of overlapping and diverse terminology can be very confusing to the reader, and special caution should be used regarding the terminology related to these methods in future reports.

Furthermore, on the topic of misleading and misused terminology, parameter-related information was inconsistent in most of the papers, e.g., pulse width was termed as the intensity [5]. In future publications, consistent and generally accepted nomenclature regarding the parameters and their purpose is recommended.

Across the studies, the treatment parameters (i.e., the settings of the machines) used were not consistent, and thus, the results are not comparable, rendering interpretation and use in a clinical context challenging. Thus, more research with a more unified selection of parameters is needed.

Considering the fact that there are several contraindications related to the modalities included in this review, it is noteworthy that most of the studies did not state the exclusion criteria.

A sufficient evidence base was not present with any of the modalities included in this systematic review. The quality of the studies varied considerably; out of the 41 studies, 14 were assessed to have a low risk of bias and 7 a moderate risk. Thus, for a few of the electrotherapies, there is at least some, although very limited, scientific basis for drawing conclusions on their effects. Several of the methods covered by this systematic review are used today in clinical practice by animal health personnel. Therefore, our review informs not only complementary and alternative practitioners, but also veterinarians and physiotherapists specialized in treating animals, on the current scientific support for various electrotherapies.

Of the 13 studies on PEMFT that we identified, 12 were assessed to have a low or moderate risk of bias. Further studies are needed to establish an appropriate list of indications and treatment parameter applications. It is also worthwhile to discuss the possible effect of using the contralateral limb for control, as a possible systemic/crossover effect of the treatment might be a confounding factor. Currently, the evidence for PEMFT is limited, at best.

Additionally, in human medicine, the evidence for favorable effects of PEMFT is very limited. There are occasional reports of beneficial effects in patients with frozen shoulder and chronic neck pain, but the studies have been of low quality [49]. For other musculoskeletal conditions, the results have generally been inconclusive. It should, however, be noted that the scientific evidence for favorable effects of transcranial field magnetic therapy in human patients with depression is sufficiently strong for the method to be recommended in clinical guidelines (e.g., [50]).

There was an overall high risk of bias in the studies on the use of NEMS in horses and dogs. The results of the study with the lowest risk of bias did not support the use of NEMS for the purpose investigated in healthy horses. Overall, for NEMS, our review did not identify any convincing scientific evidence. Regarding the common use of NEMS by physiotherapists in neurologically diseased patients, it is notable that we did not identify any scientific studies on NEMS used for this indication. In human medicine, good scientific evidence emerges from the Cochrane database for the beneficial effects of NEMS on muscle weakness in patients with severe diseases (8 RCTs; 933 patients [51]) and chronic obstructive pulmonary disease (16 RCTs; 267 patients [52]).

In human medicine, as well as clinically in veterinary medicine, TENS is traditionally used for pain management. The wide range in parameters of the electrotherapies used in the studies in companion animals is in accordance with the wide ranges used in human medicine, exemplified by frequencies ranging from 1 to 100 Hz in NEMS [53] and from below 10 Hz to 100 Hz in TENS [54].

Of the five TENS studies fulfilling the inclusion criteria of this review, only two evaluated pain as their outcome measure. However, the selection and use of outcome measures for assessing pain, specifically, were questionable in both papers. Overall, the scientific quality of studies was not high; three of the five studies were assessed to have a high risk of bias. The scientific documentation behind using TENS for pain relief or other indications in dogs and horses is therefore insufficient. That being said, no adverse reactions to the use of TENS were reported in any of the papers, unless delayed healing time relative to the other methods is considered [35].

In humans, TENS is used for acute pain such as that occurring post-surgery, during delivery, in dental interventions, and in rib fractures; this is supported by results summarized in Cochrane systematic reviews [55,56]. It is also, without strong scientific support, used for chronic pain [57].

The number of studies regarding static magnets was low, and the pathologies involved were all different. The overall conclusion is that the use of static magnets for the studied indications is not supported by the existing scientific documentation. Having said that, the intensity and exposure times to the magnet and the different types of magnet equipment were quite different across studies, hindering any comparisons or overall conclusions. The evidence status is similar in human medicine. The US National Center for Complementary and Integrative Health and the Norwegian National Center for Complementary or Alternative Medicine (NAFKAM) have both concluded that there is no scientific support for the use of static magnets for pain relief or any other pathological conditions [58].

As with the previous methods, the scientific evidence for interference is sparse. In the few papers on this method, preliminary indications of a positive treatment effect were presented in most of the studied aspects, and no harm was reported. However, it is worthwhile to note that, for humans, no systematic reviews are available from NAFKAM [58], the US National Center for Complementary and Integrative Health [59], or the Cochrane database.

Currently, PENS is clinically used in the management of equine head-shaking syndrome symptoms. We identified only two small observational studies on the topic, both with a high risk of bias. The scientific support for the use of PENS in sport and companion animals must therefore be considered very weak. When systematic literature reviews on the use of PENS in humans were searched for in databases of the U.S. National Center for Complementary and Integrative Health [59], the NAFKAM [58], and the Cochrane Library, none were found.

Due to the limited number of studies and the high risk of bias of the included studies on bioelectricity, the scientific documentation regarding the use of bioelectric modality must be summarized as very weak. However, the studies provide positive preliminary information regarding the use of the method for wound healing. When NAFKAM [58] and the Cochrane database were searched for systematic reviews regarding bioelectricity for humans, none were found.

Only two papers on short-wave diathermy with very similar materials and main parts of methodology were available for our review. The studies showed somewhat positive results, but have a high risk of bias. Considering the fact that there are no systematic reviews available on short-wave diathermy in NAFKAM [58] or in the Cochrane database, it is safe to say that the current scientific documentation for this modality and its use in animals is very weak.

For three modalities, there was only one paper per modality available that fulfilled the inclusion criteria, and two of these three studies had profound weaknesses in their methodology. Hence, the evidence regarding the use of micro-pulsed stimulation, capacitive coupled electrical stimulation, and microwave therapy is very weak. The fact that, in the report with the lowest risk bias, capacitive coupled electrical stimulation was not only of little benefit, but also tentatively associated with harmful effects, is a caveat to the further exploration of this method. Additionally, although it is obvious that the topic is not exhaustively researched, the available findings do not encourage future studies on static magnets. NAFKAM has complied information from the Cochrane Library and other published meta-analyses on the effects of static magnets on a number of musculoskeletal conditions and peripheral pain in humans. NAFKAM concluded that there is no scientific support for the favorable clinical effects of static magnets in these conditions.

The lack of studies noted in this review does not, however, necessarily indicate the redundancy of research, nor does the number of studies define the importance or the potential of the modality. The results of our systematic review indicate that a few of the electrotherapeutic methods, namely, PEMFT, TENS, and PENS, could be prioritized for further evaluation in high-quality studies. For NEMS, our review did not identify any convincing scientific evidence. Nevertheless, in light of the common use of NEMS by physiotherapists in neurologically diseased sport and companion animals and the documented effects in humans with muscular weakness associated with advanced disease, it seems reasonable to prioritize NEMS for further evaluation.

Not only the effect, but the various options for treatment parameters and therapeutic settings should be assessed further; this applies to all electrotherapies. Regarding TENS, we suggest exploring the possibilities of this modality in animals with the same indications as in humans (primarily pain) in high-quality clinical trials. Moreover, further studies of robust design are needed to provide the clinicians working with neural dysfunction and chronic pain patients with more extensive scientific documentation on the optimal use of PENS in relation to the pathologies.

## 5. Conclusions

The literature published regarding the use of various electrotherapies in horses, dogs, and cats is limited in quantity as well as quality. Most original research with the least risk of bias overall is available for PEMFT, albeit the number of studies with low risk of bias is still small. The existing literature suffers from a spectrum of indications and treatment parameters, making comparisons and drawing conclusions to support the use of these modalities in clinical practice challenging. The current scientific documentation is not sufficient to support clinical effects of electrotherapies for any clinical indication in horses, dogs or cats. Based on the results of this systematic review, high-quality evaluations of PEMFT, TENS, and PENS, and possibly also NEMS, appear to have the greatest potential to lead to conclusive results.

## Figures and Tables

**Table 1:** Electrotherapy modalities included in the review, with a short description of each.

Modality	Description
Pulsed electromagnetic field therapy (PEMFT)	Electromagnetic energy activating cell function and metabolism, e.g., stimulating synthesis of extracellular matrix structural and signaling molecules in injured tissues
Neural Electrical Muscle Stimulation (NEMS)	Pulsed current stimulating motor nerves with controlled contraction/relaxation time ratios
Transcutaneous electrical nerve stimulation (TENS)	Pulsed current stimulating sensory nerves, in humans most often used to relieve pain
Static magnet	Magnetic field activating cell function and metabolism
Interference	Crossing of two alternating currents with different frequencies
Percutaneous Electrical Neural Stimulation (PENS)	Pulsed current stimulating sensory nerves, applied by skin penetration, in humans most often used to relieve pain
Bioelectricity	Physiological electric field stimulating cell proliferation to heal injured tissue
Short-wave diathermy	High-frequency alternating electric or magnetic field producing heat, to stimulate healing
Micro-pulsed stimulation	High-voltage electric pulses, to stimulate healing
Capacitive coupled electrical stimulation	Alternating current generated field affecting bone cells
Microwave	Electromagnetic waves producing heat in tissues, to stimulate healing
